# Efficacy of Virtual Imaging for Abdominal Aortic Aneurysm Repair Complicated with Horseshoe Kidney

**DOI:** 10.3400/avd.cr.25-00111

**Published:** 2026-05-12

**Authors:** Taiki Kakiuchi, Kenichi Kamiya, Yuji Matsubayashi, Haruna Ide, Yasuo Kondo, Tomoaki Suzuki

**Affiliations:** 1 Department of Cardiovascular Surgery, Koto Memorial Hospital, Higashiomi, Shiga, Japan; 2 Department of Cardiovascular Surgery, Shiga University of Medical Science, Otsu, Shiga, Japan; 3 Department of Urology, Higashi Ohmi General Medical Center, Higashiomi, Shiga, Japan

**Keywords:** abdominal aortic aneurysm, horseshoe kidney, virtual imaging

## Abstract

Herein, we report the case of a 75-year-old male diagnosed with an infrarenal abdominal aortic aneurysm complicated by a horseshoe kidney. Coexistence of a horseshoe kidney and abdominal aortic aneurysm is a rare entity that presents a technical challenge to vascular surgeons regarding the surgical approach and reconstruction of the accessory renal arteries. Accurate and detailed preoperative studies are required to optimize surgical procedures. 3-dimensional images reconstructed from computed tomography data may be useful for visualizing complex anatomies. Open surgical repair of the aneurysm was successfully performed using virtual imaging.

## Introduction

Horseshoe kidney (HSK) is the most common congenital urological anomaly, characterized by the fusion of both kidneys during fetal development.^1)^ HSK presents with an abdominal aortic aneurysm (AAA) in 0.12% of patients,^2)^ showing several anomalies such as fused renal parenchyma and variable origins of the renal arteries or accessory branches. Various anomalies in the renal blood supply have been reported in 70% of patients with HSK.^3)^ These anomalies render surgical treatment difficult. Here, we present a surgical case of infrarenal AAA complicated by HSK that was successfully assessed using a virtual imaging technique.

## Case Report

A 75-year-old male presented with infrarenal AAA that had gradually enlarged over the past 4 years. His medical history included hypertension, hyperlipidemia, and coronary artery disease that had previously been treated with coronary artery bypass grafting. His preoperative renal function was mildly impaired, with a serum creatinine level of 1.20 mg/dL and an estimated glomerular filtration rate (eGFR) of 46 mL/min/1.73 m^2^.

**Fig. 1:**
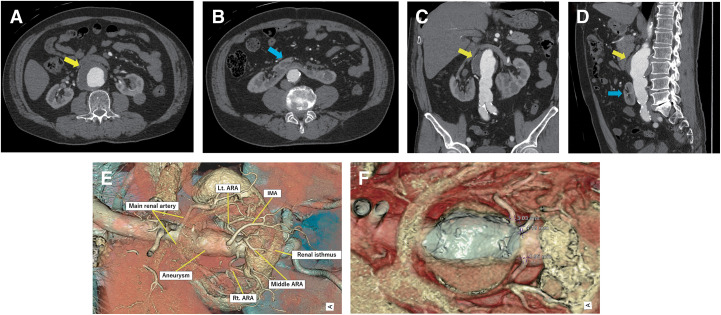
Preoperative CTA image showing an infrarenal AAA (yellow arrow) with a coexistent HSK (blue arrow). (**A**) Axial images showing the maximum diameter (56 mm) of the AAA and (**B**) HSK, in which the inferior poles were fused on the ventral side of the AAA. (**C**) Coronal view of AAA with HSK. (**D**) Sagittal view of AAA with HSK. (**E**) 3-dimensional volume-rendered image showing the 3 ARAs and IMA originating from the distal aneurysmal sac, and ARAs originating from the same aortic level. The middle ARA supplied the renal isthmus. The pattern of the horseshoe kidney with the ARAs is type III in Eisendrath’s classification. (**F**) Transverse view showing the diameters of the ARAs supplying the left, middle, and right renal portions (3.03, 4.18, and 1.95 mm, respectively). Lt: left; Rt: right; CTA: computed tomography angiography; AAA: abdominal aortic aneurysm; HSK: horseshoe kidney; ARA: accessory renal artery; IMA: inferior mesenteric artery

**Fig. 2:**
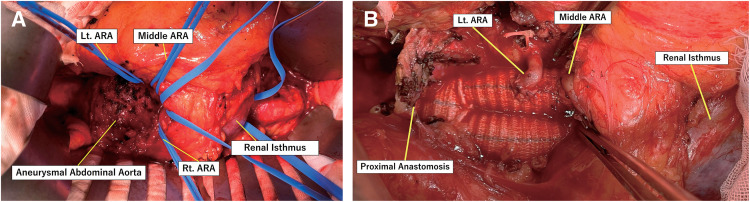
(**A**) Intraoperative image showing the AAA and renal isthmus of the horseshoe kidney lying anterior to the AAA. 3 ARAs were secured with surgical tape, and the renal isthmus was carefully mobilized with surgical tape after separating the isthmus from the aneurysmal wall. (**B**) The proximal anastomosis and reimplantation of the left and middle ARAs. Lt: left; Rt: right; AAA: abdominal aortic aneurysm; ARA: accessory renal artery

**Fig. 3:**
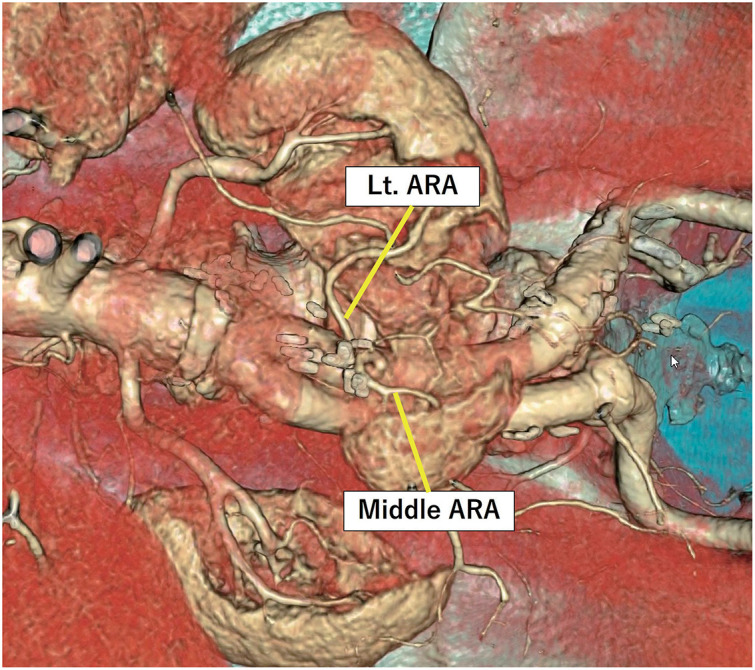
Postoperative computed tomography scan showing patency of 2 reconstructed ARAs and good enhancement of the renal parenchyma. Lt: left; ARA: accessory renal artery

Preoperative contrast-enhanced computed tomography angiography (CTA) revealed infrarenal AAA (56 mm in diameter) and HSK, in which the inferior poles were fused on the ventral side of the AAA (**Fig. 1A**–**1D**). Although the patient was indicated for operative treatment, 2-dimensional multiplanar CTA images provided limited preoperative information for planning optimal surgical strategies, particularly focusing on the vascular geometries associated with HSK. Therefore, we processed the CTA images using a virtual imaging software (Vesalius 3D; PS-Tech, Amsterdam, the Netherlands) to visualize the detailed abdominal aortic anatomy. CTA was performed using a 64-row multidetector computed tomography (CT) scanner (Revolution GSI; GE Healthcare, Chicago, IL, USA) with 1-mm slice collimation. The Digital Imaging and Communications in Medicine (DICOM) data were transferred to Vesalius3D software (version 2.12; PS-Tech) for post-processing. Volume-rendered (VR) images were generated using a graphics processing unit-based ray-casting algorithm, allowing high-resolution visualization of the aorta, HSK, and associated vasculature. The 3-dimensional VR (3D-VR) image revealed that the 2 main renal arteries originated from the relatively short neck of the proximal aorta, and the 3 accessory renal artery (ARA) branches and inferior mesenteric artery (IMA) originated from the distal aneurysmal sac. All ARAs were located at the same aortic level, and the middle ARA branch supplied the renal isthmus (**Fig. 1E**). The diameters of the ARAs supplying the left, middle, and right renal portions were 3.03, 4.18, and 1.95 mm, respectively (**Fig. 1F**). In addition, the renal veins and ureters were evaluated on delayed-phase CT images. Both structures followed a typical course without displacement related to the HSK, and their positions were confirmed to have no effect on the planned surgical field. To further illustrate the 3D anatomical view, virtual images are shown in **Supplementary Video**. Based on these anatomical features, an open repair approach was considered, whereas endovascular aortic repair requires occlusion of all ARAs and the IMA, which may cause a potential risk of type II endoleaks. We also planned to preserve the left and middle ARAs with diameters >2 mm to avoid postoperative renal dysfunction. The operative procedure was performed via median laparotomy. Consistent with the preoperative imaging, intraoperative findings revealed that the renal isthmus of the HSK appeared anterior to the aneurysm, and 3 ARA branches and IMA were identified precisely at the same locations as the corresponding preoperative 3D-VR images (**Fig. 2A**). We carefully mobilized the renal isthmus with surgical tape and dissected all branched vessels after separating the isthmus from the aneurysmal wall. After systemic heparinization, the proximal aorta and distal common iliac arteries were cross-clamped, and the secured branched vessels were clamped. After the aneurysm sac was opened, the intraluminal thrombus was removed, and the proximal aorta and bilateral distal iliac arteries were carefully trimmed and replaced using an 18 × 11-mm bifurcated Dacron graft (J graft; Japan Lifeline, Tokyo, Japan). Next, the left and middle ARAs were anastomosed to the left leg of the prosthetic graft (**Fig. 2B**). Finally, the right ARA (<2 mm in diameter) and IMA were ligated. The total operating time was 4 h and 46 min, including 1 h and 29 min of total aortic cross-clamping time, with an intraoperative blood loss of 1580 mL. The patient’s postoperative clinical course was uneventful. Postoperative renal function temporarily deteriorated but improved to the preoperative eGFR level. The patient was transferred from the intensive care unit on postoperative day 1 and was discharged on postoperative day 8. Postoperative CT after 6 months showed patency of the 2 reconstructed ARAs and good enhancement of the renal parenchyma (**Fig. 3**).

## Discussion

Surgical treatment of AAA with HSK can be technically challenging owing to its unique anatomical features, which are mainly associated with variable locations of the renal isthmus and vascular supply to the renal parenchyma. Therefore, accurate preoperative assessment for the management of renal isthmus and arterial variations may play an important role in optimizing surgical strategies.

An abnormal vascular anatomy is common in HSK and often includes ARAs arising from the aorta, IMA, or iliac arteries. A commonly used classification system categorizes these vascular patterns into 5 types,^4)^ with types I and II accounting for approximately half of all cases. Type I has 1 renal artery supplying each kidney; type II includes an additional aortic branch supplying the isthmus; type III includes an additional renal artery supplying each side; type IV includes 2 renal arteries on each side, with some arising from the iliac arteries or the isthmus branch; and type V involves multiple renal arteries originating from the aorta, mesenteric arteries, and iliac arteries. In our case, 2 renal arteries were present on each side along with an isthmic branch, corresponding to type III. The surgical approach for AAA with HSK is associated with some technical difficulties and has several technical options. The first involves approaching the aneurysm through a midline incision or a left retroperitoneal approach.^4)^ The midline approach allows wide exposure of the aneurysm sac, the renal isthmus, and both iliac arteries. Conversely, the left retroperitoneal approach has the advantage of avoiding interference with the urinary tract and renal isthmus and allowing visualization of the ARAs.^4)^ The second is a surgical approach involving division of the renal isthmus, which allows complete isolation of the HSK and full exposure of the aneurysm. However, this technique carries potential risks of urinary leaks, infections, bleeding, and diffuse renal ischemia.^4)^ Routine isthmus resection can be avoided unless the renal isthmus prevents surgical exposure and subsequent anastomotic procedures. In the present case, preoperative 3D-VR images indicated that we could secure the ARAs using caudal traction of the renal isthmus without division of the renal isthmus. Therefore, a midline approach was selected, and we achieved a satisfactory surgical field around the renal isthmus, the ARAs, and both iliac arteries.

Among the 3 ARAs, the left and middle vessels were reconstructed, whereas the right ARA was not preserved. Although reconstruction of the right ARA was technically feasible, it was avoided for 2 reasons. First, the renal isthmus overlapped the vessel, making exposure difficult and increasing the risk of prolonged dissection and extended aortic clamping time. Second, the vessel diameter was <2 mm, and preservation was unlikely to contribute substantially to renal perfusion. Canova et al.^3)^ reported that ARAs <2 mm can be ligated without a significant risk of renal complications, and another report on endovascular AAA repair in HSK suggested that vessels <3 mm do not require reconstruction.^5)^ These findings support our decision to ligate the right ARA. Postoperatively, the patient experienced no renal complications and recovered uneventfully, further validating this approach. In small-caliber arterial reconstructions, published data remain limited; however, evidence from kidney transplantation suggests that reconstruction of ARAs measuring 2–4 mm can achieve acceptable patency, with a reported rate of 77.8%, and no significant correlation between vessel diameter and patency.^6)^ These findings support the feasibility of reconstructing small arteries when technically achievable.

Virtual imaging offers a relatively intuitive exploration of the anatomy from any angle, comprehensive orientation with the surrounding structures, and measurement of complex cardiovascular structures. In this case, virtual imaging directly influenced the surgical strategy and contributed to perioperative safety rather than serving as a purely descriptive adjunct. First, preoperative 3D-VR images demonstrated that all 3 ARAs originated at the same aortic level and could be secured by caudal traction of the renal isthmus without division. Because isthmus division is associated with risks such as urinary leakage, bleeding, infection, and renal ischemia, the ability to confidently avoid this maneuver was considered a meaningful advantage in reducing potential complications. Second, virtual imaging enabled a precise preoperative identification of reconstructable ARAs, allowing selective revascularization of the left and middle branches while safely ligating the small right ARA. This targeted planning minimized unnecessary dissection and helped prevent prolonged aortic clamping time. Finally, the intraoperative findings corresponded exactly with the virtual images, reducing uncertainty during exposure and reconstruction. Collectively, these factors suggest that virtual imaging played a causal role in optimizing operative strategy and enhancing procedural safety in this anatomically complex case.

Building on recent advances in 3D visualization that enable the depiction of complex anatomical structures with VR tissue information,^7)^ this platform provides stereoscopic depth perception and a realistic spatial understanding of the anatomy. These complementary techniques allow virtual imaging to reproduce a 3D operative perspective that closely matches the surgeon’s field of view, enabling a clearer appreciation of depth relationships and more confident identification of anatomical landmarks than conventional 3D-VR CTA. The efficacy of this technology has previously been reported for cases of anatomically complicated thoracic aortic aneurysm^8)^ and congenital aortic root anomaly.^9)^ In this case, the preoperative 3D-VR CTA image clearly showed the position of the bilateral kidneys, the morphology of the renal isthmus, the relationship between the renal parenchyma and aortic wall, and the dimensions and properties of the ARAs. By integrating these details into a virtual operative environment, the surgeon can simulate exposure, clamping, and reconstruction strategies, thereby reducing intraoperative uncertainty and enhancing procedural safety. This information helps optimize the surgical procedure involved in the reconstruction of ARAs and helps avoid intraoperative and postoperative complications. This technology may be used in addition to conventional CTA for anatomically complicated AAA cases with HSK. Further clinical case studies are warranted to investigate the effectiveness of this technique in facilitating operative success in AAA with HSK and to evaluate its potential as a useful tool for surgical training.

## Conclusion

Surgical treatment of AAA with HSK can be technically challenging owing to its complicated anatomical features. This 3D-VR imaging technique enabled us to assess the anatomy of AAA complicated by HSK easily and quickly, allowing accurate preoperative planning. This technology can provide outstanding assistance for the optimization of anatomically complicated vascular surgeries.

## Supplementary Materials

Supplementary Video Three-dimensional volume-rendered images show detailed anatomical information, such as the position of the bilateral kidneys, morphology of the renal isthmus, relationship between the renal parenchyma and aortic wall, dimensions and properties of ARAs, and other surrounding structures.
